# Current Smoking and Prognosis After Acute ST-Segment Elevation Myocardial Infarction: New Pathophysiological Insights

**DOI:** 10.1016/j.jcmg.2018.05.022

**Published:** 2019-06

**Authors:** Caroline Haig, David Carrick, Jaclyn Carberry, Kenneth Mangion, Annette Maznyczka, Kirsty Wetherall, Margaret McEntegart, Mark C. Petrie, Hany Eteiba, Mitchell Lindsay, Stuart Hood, Stuart Watkins, Andrew Davie, Ahmed Mahrous, Ify Mordi, Nadeem Ahmed, Vannesa Teng Yue May, Ian Ford, Aleksandra Radjenovic, Paul Welsh, Naveed Sattar, Keith G. Oldroyd, Colin Berry

**Affiliations:** a Robertson Centre for Biostatistics, University of Glasgow, Glasgow, United Kingdom; b British Heart Foundation Glasgow Cardiovascular Research Centre, Institute of Cardiovascular and Medical Sciences, University of Glasgow, Glasgow, United Kingdom; c West of Scotland Heart and Lung Centre, Golden Jubilee National Hospital, Clydebank, United Kingdom

**Keywords:** cigarette smoking, magnetic resonance imaging, microcirculation, myocardial hemorrhage, myocardial infarction, prognosis, CI, confidence interval, IMR, index of microcirculatory resistance, LV, left ventricular, MACE, major adverse cardiovascular event(s), MI, myocardial infraction, MRI, magnetic resonance imaging, MVO, microvascular obstruction, NT-proBNP, N-terminal pro–B-type natriuretic peptide, OR, odds ratio, PCI, percutaneous coronary intervention, STEMI, ST-segment elevation myocardial infarction

## Abstract

**Objectives:**

The aim of this study was to mechanistically investigate associations among cigarette smoking, microvascular pathology, and longer term health outcomes in patients with acute ST-segment elevation myocardial infarction (MI).

**Background:**

The pathophysiology of myocardial reperfusion injury and prognosis in smokers with acute ST-segment elevation MI is incompletely understood.

**Methods:**

Patients were prospectively enrolled during emergency percutaneous coronary intervention. Microvascular function in the culprit artery was measured invasively. Contrast-enhanced magnetic resonance imaging (1.5-T) was performed 2 days and 6 months post-MI. Infarct size and microvascular obstruction were assessed using late gadolinium enhancement imaging. Myocardial hemorrhage was assessed with T2* mapping. Pre-specified endpoints included: 1) all-cause death or first heart failure hospitalization; and 2) cardiac death, nonfatal MI, or urgent coronary revascularization (major adverse cardiovascular events). Binary logistic regression (odds ratio [OR] with 95% confidence interval [CI]) with smoking status was used.

**Results:**

In total, 324 patients with ST-segment elevation MI were enrolled (mean age 59 years, 73% men, 60% current smokers). Current smokers were younger (age 55 ± 11 years vs. 65 ± 10 years, p < 0.001), with fewer patients with hypertension (52 ± 27% vs. 53 ± 41%, p = 0.007). Smokers had better TIMI (Thrombolysis In Myocardial Infarction) flow grade (≥2 vs. ≤1, p = 0.024) and ST-segment resolution (none vs. partial vs. complete, p = 0.010) post–percutaneous coronary intervention. On day 1, smokers had higher circulating C-reactive protein, neutrophil, and monocyte levels. Two days post-MI, smoking independently predicted infarct zone hemorrhage (OR: 2.76; 95% CI: 1.42 to 5.37; p = 0.003). After a median follow-up period of 4 years, smoking independently predicted all-cause death or heart failure events (OR: 2.20; 95% CI: 1.07 to 4.54) and major adverse cardiovascular events (OR: 2.79; 95% CI: 2.30 to 5.99).

**Conclusions:**

Smoking is associated with enhanced inflammation acutely, infarct-zone hemorrhage subsequently, and longer term adverse cardiac outcomes. Inflammation and irreversible myocardial hemorrhage post-MI represent mechanistic drivers for adverse long-term prognosis in smokers. (Detection and Significance of Heart Injury in ST Elevation Myocardial Infarction. [BHF MR-MI]; NCT02072850)

## Graphical abstract

**Figure:**
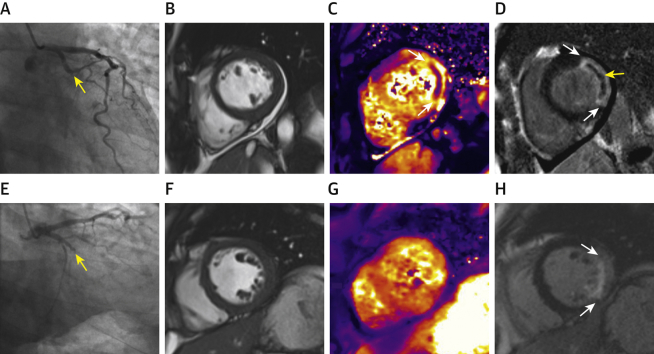

Cigarette smoking is persistently common 1, 2 and causal in the pathophysiology of acute myocardial infarction (MI) 3, 4, 5, 6. An apparent paradoxical relationship has been described between smoking and prognosis after acute MI, with lower crude rates of adverse cardiac events reported 7, 8, 9, 10, 11, 12. Confounders, including younger age and fewer vascular risk factors, may explain the smoker’s paradox 6, 7, 8, 9, 10, 11, 12. Moreover, the duration of follow-up in many studies was ≤12 months, and those studies with longer follow-up identified smoking as an adverse prognostic factor 13, 14, 15, implying the risk may operate in the longer term post-MI.

The pathophysiology of myocardial reperfusion injury in smokers with acute ST-segment elevation MI (STEMI) is incompletely understood. Higher epicardial coronary flow rates after primary percutaneous coronary intervention (PCI) have been reported in current smokers versus nonsmokers (10). Studies of smoking and microvascular pathology, using cardiac magnetic resonance (CMR), have reported conflicting results 12, 16, specifically pertaining to myocardial hemorrhage (an independent predictor of adverse outcome post-MI) 17, 18. The MRI method in previous studies was not specific for detecting myocardial hemorrhage, and information on health outcomes was limited to 12-month follow-up (12) or was unavailable (16). T2* mapping has emerged as a specific technique for detecting myocardial hemorrhage post-MI (18), with potential to resolve this controversy. Further insights into the effect of smoking on myocardial reperfusion injury could be provided by invasive microcirculatory assessment in the culprit coronary artery, using index of microcirculatory resistance (IMR), which is associated with infarct size, left ventricular (LV) pathology, and health outcomes 19, 20, 21, 22, 23, 24.

We aimed to prospectively investigate associations between smoking, microvascular pathology and longer term health outcomes in patients with acute STEMI. We hypothesized that current smoking in patients with acute STEMI would be associated with acute microvascular dysfunction revealed by IMR and progressive hemorrhagic transformation within the infarct zone revealed by T2* MRI 2 days post-MI. We also hypothesized that smoking would be an independent predictor of adverse longer term health outcomes.

## Methods

### Study population

We performed a prospective cohort study at a regional cardiac center from July 2011 to November 2012. Patients provided written informed consent to undergo diagnostic guidewire-based assessment after reperfusion, then MRI 2 days and 6 months later and follow-up for health outcomes in the longer term. Patients were eligible if they had indications for primary PCI or thrombolysis for acute STEMI (25). Exclusion criteria were standard contraindications to MRI. Current smoking status was defined as use of ≥100 cigarettes by a patient in his or her lifetime who currently routinely smoked cigarettes (26). Primary PCI and secondary prevention measures, including cardiac rehabilitation with risk factor management, were implemented according to contemporary guidelines (25). The study was approved by the National Research Ethics Service (10-S0703-28). The ClinicalTrials.gov identifier is NCT02072850.

### IMR in the culprit coronary artery

A coronary guidewire with a pressure and temperature sensor (St. Jude Medical, St. Paul, Minnesota) was used to measure IMR in the culprit coronary artery at the end of primary or rescue PCI. The guidewire was calibrated outside the body, equalized with aortic pressure at the ostium of the guide catheter, and then advanced to the distal third of the culprit artery. IMR, an invasive measure of microvascular resistance, is defined as distal coronary pressure multiplied by mean transit time of a bolus of saline at room temperature, during maximal coronary hyperemia (23). Hyperemia was induced by 140 μg/kg/min of intravenous adenosine preceded by an intracoronary bolus of 200 μg of nitrate. We previously found IMR to be highly repeatable when assessed by duplicate measurements 5 min apart in 12 consecutive patients with STEMI at the end of PCI (20).

### Electrocardiography

Twelve-lead electrocardiograms were obtained before coronary reperfusion and 60 min afterward. ST-segment resolution assessed 60 min after reperfusion was compared with the baseline electrocardiogram before reperfusion and was expressed as complete (70%), incomplete (30% to <70%), or none (30%).

### Coronary angiographic acquisition and analyses

Coronary angiograms were acquired during usual care with a cardiac catheter laboratory radiograph (Innova, GE Healthcare, Little Chalfont, United Kingdom) and information technology equipment (Centricity, GE Healthcare). Angiograms were analyzed by trained observers (J.C., V.T.Y.M.) who were blinded to other clinical and MRI data. TIMI (Thrombolysis In Myocardial Infarction) coronary flow grade (27) and frame count (28) were assessed at initial angiography and at the end of the procedure. TIMI myocardial perfusion grade (29) was assessed at the end of the procedure (Supplemental Appendix).

### Laboratory analyses

The blood samples and analyses of hematology and biochemistry, including serum troponin T, C-reactive protein, and N-terminal pro–B-type natriuretic peptide (NT-proBNP) are described in the Supplemental Appendix.

### Cardiac MRI

**Figure 1:**
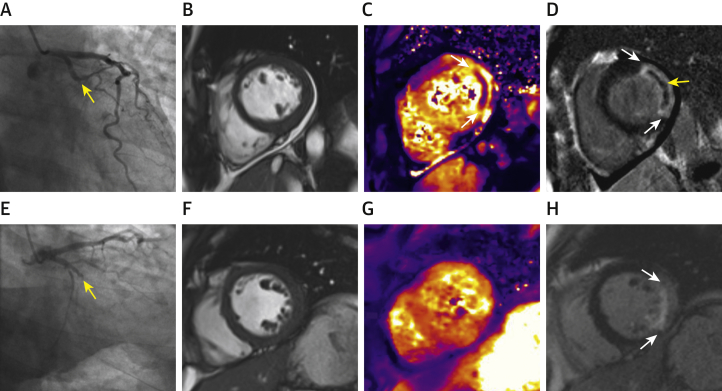
Smoking and Myocardial Hemorrhage After Acute ST-Segment Elevation Myocardial Infarction Current smoker **(A to D)** and nonsmoker **(E to H)** who underwent primary percutaneous coronary intervention (PCI) for lateral ST-segment elevation myocardial infarction. Both had TIMI (Thrombolysis in Myocardial Infarction) flow grade 3 post-PCI. Antithrombotic therapies, including aspirin, clopidogrel, and unfractionated heparin, were similar for both patients. Magnetic resonance imaging (MRI) was performed 2 days and 6 months later. **(A to D)** Imaging from a 58-year-old male current smoker. Symptom-to-balloon time was 8.5 h. Angiography revealed a proximally occluded circumflex coronary artery **(yellow arrow, A)**. Two days later, myocardial hemorrhage was revealed by T2* mapping **(white arrow, C)**. Late gadolinium enhancement revealed transmural infarction of the lateral left ventricular (LV) wall **(white arrows, D)** associated with microvascular obstruction (MVO) **(yellow arrow, D)**. Baseline infarct size was 32.3%, LV ejection fraction 55%, and LV end-diastolic volume indexed to body surface area (LVEDVi) 83.8 ml/m^2^. Six months later, infarct size was 31.2% and LVEDVi 84.8ml/m^2^, consistent with adverse remodeling. **(E to H)** Imaging from a 48-year-old male nonsmoker. Symptom-to-balloon time was 9.3 h. Angiography revealed a proximally occluded circumflex coronary artery **(yellow arrow, E)**. Two days later, there was no evidence of myocardial hemorrhage **(G)** or MVO within the infarct zone **(H)** on MRI. Baseline infarct size was 25.3%, LV ejection fraction 51%, and LVEDVi 78.4 ml/m^2^. Six months later, infarct size was 15.2% and LVEDVi 72.7 ml/m^2^.

MRI was used to provide reference data on LV function, pathology, and surrogate outcomes (Figure 1). MRI was performed using a Siemens MAGNETOM Avanto (Siemens Healthcare, Erlangen, Germany) 1.5-T scanner with a 12-element phased-array cardiac surface coil. The imaging protocol 18, 21, 22, 30 (Supplemental Appendix) included cine MRI with steady-state free precession, T2 mapping 31, 32, T2* mapping (18), and delayed-enhancement phase-sensitive inversion recovery pulse sequences (33). The scan acquisitions were spatially coregistered and also included different slice orientations to enhance diagnostic confidence.

### Imaging analyses

The MRI analyses are described in detail in the Supplemental Appendix. The results of infarct characteristics are reported for the whole of the left ventricle.

### Infarct size, microvascular obstruction, and myocardial hemorrhage

The presence of acute infarction was established on the basis of abnormalities in cine wall motion, rest first-pass myocardial perfusion, and delayed-enhancement imaging in 2 imaging planes. The myocardial mass of late gadolinium (grams) was quantified using computer-assisted planimetry, and the territory of infarction was delineated using a signal intensity threshold of >5 SDs above a remote reference region and expressed as a percentage of total LV mass 18, 21, 22, 30, 34.

Microvascular obstruction (MVO) was defined as a dark zone on late gadolinium enhancement imaging 1, 3, and 5 min post–contrast injection that remained present within an area of late gadolinium enhancement at 15 min. On the T2* MRI maps, a region of reduced signal intensity within the infarcted area with a T2* value of <20 ms 18, 35 was considered to confirm the presence of myocardial hemorrhage.

### Myocardial edema and salvage

The extent of myocardial edema was defined as LV myocardium with pixel values >2 SDs from remote myocardium 36, 37, 38. Myocardial salvage was calculated by subtraction of percentage infarct size from percentage area at risk, as reflected by the extent of edema 20, 36, 37, 38, 39. The myocardial salvage index was calculated by dividing the myocardial salvage area by the initial area at risk.

### LV remodeling

An increase of ≥20% in LV end-diastolic volume at 6 months from baseline was taken to reflect adverse LV remodeling (40).

### Pre-specified health outcomes

The primary composite outcome was all-cause death or first heart failure event following the initial hospitalization. The second composite outcome was major adverse cardiovascular events (MACE) including cardiac death, nonfatal MI, and urgent coronary revascularization. These outcomes were independently assessed by cardiologists blind to the baseline findings (Supplemental Appendix) (41).

### Statistical analyses

The sample size calculation and statistical methods are described in the Supplemental Appendix. Differences in continuous variables between groups were assessed using the Student *t*-test or analysis of variance for parametric data and the Mann-Whitney *U* test or Kruskal-Wallis *H* test for nonparametric data. Differences in categorical variables were assessed using a chi-square or Fisher exact test. Univariate and multivariate associations were assessed using binary logistic regression or linear regression as appropriate. Binary logistic models were compared using Harrel’s C statistic. Logistic regression (odds ratio [OR] and 95% confidence interval [CI]) was used to identify clinical predictors (patient characteristics and MRI findings) of all-cause death or heart failure events. Statistical analyses were performed using R version 2.15.1 (R Foundation for Statistical Computing, Vienna, Austria) or SAS version 9.3 (SAS Institute, Cary, North Carolina). A p value <0.05 represented statistical significance.

## Results

### Patient characteristics

**Figure 2:**
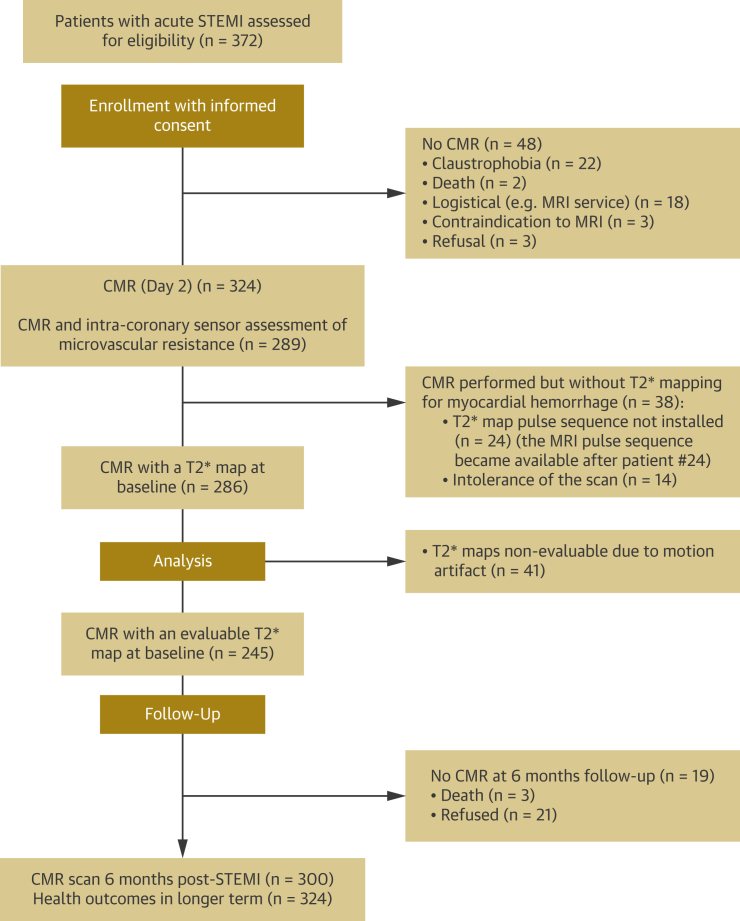
Flow Diagram Consolidated Standards of Reporting Trials flow diagram of the study. CMR = cardiac magnetic resonance; MRI = magnetic resonance imaging; STEMI = ST-segment elevation myocardial infarction.

Of 372 consecutive patients with acute STEMI who were assessed for eligibility, 324 (87%) (mean age 59 ± 12 years, 237 [73%] men, 196 [60%] smokers) were enrolled (Table 1, Figure 2). Reasons for not being enrolled are detailed in Figure 2. Compared with nonsmokers, current smokers were younger and had fewer cardiovascular risk factors (Table 1). The distribution of the infarct-related artery differed between smokers and nonsmokers, whereas the biochemical size of infarction was similar.

**Table 1:** Clinical and Angiographic Characteristics of 324 Patients With ST-Segment Elevation Myocardial Infarction Categorized According to Smoking Status at Initial Presentation

	All Patients (N = 324)	Nonsmokers (n = 128 [40%])	Current Smokers (n = 196 [60%])	p Value
Age, yrs	59 ± 12	65 ± 10	55 ± 11	<0.001 (t)
Male	237 (73)	98 (77)	139 (71)	0.305
BMI, kg/m^2^	28.8 ± 4.8	29.1 ± 4.6	28.6 ± 4.8	0.346 (t)
Hypertension	105 (32)	53 (41)	52 (27)	0.007
Hypercholesterolemia	94 (29)	44 (34)	50 (26)	0.103
Diabetes mellitus∗	34 (11)	14 (11)	20 (10)	0.854
Previous myocardial infarction	25 (8)	11 (9)	14 (7)	0.673
Previous PCI	18 (6)	8 (6)	10 (5)	0.805
Presenting characteristics				
Heart rate, beats/min	78 ± 17	77 ± 16	78 ± 18	0.518 (t)
Systolic blood pressure, mm Hg	135 ± 25	137 ± 23	134 ± 25	0.264 (t)
Symptom-to-reperfusion time, min	174 (120–315)	176 (120–307)	171 (122–324)	0.984 (MW)
Killip class at presentation†				
I	233 (72)	100 (78)	133 (68)	
II	68 (21)	21 (16)	47 (24)	0.138
III/IV	23 (7)	7 (6)	16 (8)	
ST-segment resolution post-PCI				
Complete, ≥70%	148 (46)	46 (36)	102 (52)	
Incomplete, 30% to <70%	127 (39)	57 (45)	70 (36)	0.010
None, ≤30%	48 (15)	25 (20)	23 (12)	
Reperfusion strategy				
Primary PCI	302 (93)	122 (95)	180 (92)	
Rescue PCI (failed thrombolysis)	14 (4)	4 (3)	10 (5)	0.497
Successful thrombolysis	8 (3)	2 (2)	6 (3)	
Coronary angiography				
Number of diseased arteries‡				
1	174 (54)	64 (50)	110 (56)	
2	99 (31)	41 (32)	58 (30)	0.635
3	45 (14)	21 (16)	24 (12)	
Culprit artery				
LM	6 (2)	2 (2)	4 (2)	
LAD	121 (37)	50 (39)	71 (36)	
LCx	59 (18)	15 (12)	44 (22)	0.045
RCA	144 (44)	63 (49)	81 (41)	
TIMI coronary flow grade pre-PCI				
0/1	236 (73)	93 (73)	143 (73)	
2/3	88 (27)	35 (27)	53 (27)	1.000
TIMI coronary flow grade post-PCI				
0/1	4 (1)	4 (3)	0 (0)	
2/3	320 (99)	124 (97)	196 (100)	0.024
TIMI frame count post-PCI	15.9 (10.0-24.3)	15.7 (10.0–24.0)	16.0 (9.9–24.7)	0.631 (MW)
TIMI blush grade post-PCI				
0	70 (23)	26 (21)	44 (24)	
1	17 (6)	7 (6)	10 (5)	0.901
2	157 (51)	65 (53)	92 (49)	
3	65 (21)	24 (20)	41 (22)	
Culprit lesion, percentage residual stenosis	12.4 (5.4)	12.4 (5.6)	12.4 (5.2)	0.997 (t)
Index of microvascular resistance§	1.6 (1.1–2.1)	27 (16–47)	22 (15–41)	0.062
Aspiration thrombectomy	236 (73)	92 (72)	144 (74)	0.799
Glycoprotein IIb/IIIa inhibitor	297 (92)	118 (92)	179 (91)	0.840
Medical therapy at discharge				
ACE inhibitor or ARB	320 (99)	127 (98)	193 (99)	0.579
Beta-blocker	308 (95)	121 (95)	187 (95)	0.795
Statin	324 (100)	128 (100)	196 (100)	1.00
Antiplatelet therapy				
Aspirin	323 (99.7)	128 (100.0)	195 (99.5)	1.00
Clopidogrel	321 (99.1)	127 (99.2)	194 (99.0)	1.00
Initial blood results on admission				
Creatinine, μg/l	77.8 ± 18.9	83.2 ± 21.4	74.3 ± 16.2	<0.001 (t)
C-reactive protein, mg/l§	4 (2–7)	3 (2–7)	4 (2–8)	0.035 (MW)
Interleukin-6, pg/ml	6.8 (4.4–10.8)	7.8 (4.6–12.3)	6.4 (4.4–10.6)	0.531 (MW)
Neutrophil count, ×10^6^/l	9.1 (7.2–11.6)	8.3 (6.7–10.4)	9.6 (7.9–12.1)	<0.001 (MW)
Monocyte count, ×10^6^/l	0.8 (0.6–1.0)	0.7 (0.6–0.9)	0.9 (0.7–1.1)	<0.001 (MW)
NT-proBNP, pg/l	864 (345–1,637)	1,040 (529–1,860)	646 (300–1,388)	0.022 (MW)
Troponin T, ng/l§	1,710 (110–5,099)	1,496 (82–4,410)	1,945 (178–5,133)	0.265 (MW)

Values are mean ± SD, n (%), or median (interquartile range). The p values were obtained from Student *t*-tests (t), Mann-Whitney *U* tests (MW), or Fisher exact tests. TIMI flow grades pre- and post-PCI were grouped as 0/1 versus 2/3 for this analysis.

ACE = angiotensin-converting enzyme; ARB = angiotensin receptor blocker; BMI = body mass index; LAD = left anterior descending coronary artery; LCx = left circumflex coronary artery; LM = left main coronary artery; NT-proBNP = N-terminal pro–brain natriuretic peptide; PCI = percutaneous coronary intervention; RCA = right coronary artery; TIMI = Thrombolysis In Myocardial Infarction.

∗ Diabetes mellitus was defined as a history of diet-controlled or treated diabetes.

† Killip classification of heart failure after acute myocardial infarction: class I, no heart failure; class II, pulmonary rales or crepitations, a third heart sound, and elevated jugular venous pressure; class III, acute pulmonary edema; class IV, cardiogenic shock.

‡ Multivessel coronary artery disease was defined according to the number of stenoses of at least 50% of the reference vessel diameter, by visual assessment, and whether or not there was left main stem involvement.

§ C-reactive protein was available in 316 subjects, and troponin T was available in 313 subjects. IMR was available in 283 subjects.

### Culprit artery blood flow and reperfusion injury

At the end of the emergency PCI procedure, TIMI flow grade in the culprit coronary artery was higher (≥2 vs. ≤1) in smokers than nonsmokers (p = 0.024), whereas TIMI blush grade and frame count were similar between groups. At 60 min post-reperfusion, ST-segment resolution was achieved more often in smokers than nonsmokers (p = 0.01). IMR tended to be lower in smokers (22 [interquartile range: 15 to 41] vs. 27 [interquartile range: 16 to 47], p = 0.062), although the difference was not statistically significant.

### Systemic inflammation and NT-proBNP

On day 1, circulating C-reactive protein, neutrophil, and monocyte levels were higher in current smokers, whereas NT-proBNP concentrations were lower (Table 1).

### Cardiac MRI findings

A total of 324 patients underwent MRI 2.1 ± 1.8 days after hospitalization, and 295 patients (91%) underwent follow-up MRI at 6 months (Table 2, Figure 2). Case examples are shown in Figure 1.

**Table 2:** Cardiac Magnetic Resonance Imaging Findings at 2 Days and 6 Months Post-Reperfusion in 324 Patients With ST-Segment Elevation Myocardial Infarction Categorized According to Smoking Status

	All Patients (n = 324)	Nonsmokers (n = 128 [40%])	Current Smokers (n = 196 [60%])	p Value
CMR findings 2 days post-MI (n = 324)				
LV ejection fraction, %	55 ± 10	55 ± 10	55 ± 10	0.802 (t)
LVEDV, ml				
Men	161 ± 33	156 ± 28	165 ± 36	0.051 (t)
Women	125 ± 25	122 ± 27	127 ± 24	0.423 (t)
LVESV, ml				
Men	75 ± 27	72 ± 24	78 ± 28	0.117 (t)
Women	55 ± 18	55 ± 19	55 ± 18	0.856 (t)
LV mass, g				
Men	145 ± 33	139 ± 27	149 ± 36	0.016 (t)
Women	99 ± 23	96 ± 25	100 ± 22	0.711 (t)
Edema and infarct characteristics				
Myocardial edema, percentage LV mass	32 ± 12	32 ± 12	32 ± 12	0.663 (t)
T2 relaxation times (ms) in regions of interest				
Infarct zone	62.9 ± 5.1	63.3 ± 5.0	62.6 ± 5.2	0.224 (t)
Infarct core	53.9 ± 4.8	54.1 ± 4.7	53.7 ± 5.0	0.570 (t)
Remote zone T2	49.7 ± 2.1	49.5 ± 2.0	49.9 ± 2.1	0.176 (t)
Infarct size, percentage LV mass	16 ± 7, 27	19 ± 7, 27	15 ± 7, 28	0.752 (MW)
Myocardial salvage index, percentage of LV mass	63 ± 24	64 ± 23	62 ± 25	0.678 (t)
Late microvascular obstruction	164 (51)	62 (48)	102 (52)	0.570
Late microvascular obstruction, percentage LV mass	0.2 (0.0 to 3.5)	0.0 (0.0 to 3.3)	0.3 (0.0 to 3.8)	0.572 (MW)
Myocardial hemorrhage	101 (41)	31 (34)	70 (46)	0.081
CMR findings 6 months post-MI (n = 295)				
LV ejection fraction at 6 months, %	63 (57 to 69)	63 (57 to 68)	63 (56 to 69)	0.780 (MW)
LVESV at 6 months, ml				
Men	62 (44 to 79)	60 (42 to 75)	64 (48 to 82)	0.121 (MW)
Women	43 (33 to 58)	49 (39 to 60)	41 (33 to 54)	0.119 (MW)
Change in LVEDV at 6 months from baseline, ml				
Men	7 (−7 to 21)	7 (−8 to 18)	6 (−7 to 22)	0.687 (MW)
Women	1 (−12 to 9)	3 (−11 to 9)	−2 (−13 to 10)	0.639 (MW)

Values are mean ± SD, n (%), or median (interquartile range). Area at risk was measured using T2 mapping. The p values were obtained using Student *t*-tests (t), Mann-Whitney *U* tests (MW), or Fisher exact tests. LV ejection fraction was missing in 29 subjects at follow-up. LVEDV at follow-up was missing in 16 men and 8 women. T2* imaging for myocardial hemorrhage was available in 245 subjects.

CMR = cardiac magnetic resonance; LV = left ventricular; LVEDV = LV end-diastolic volume; LVESV = left ventricular end-systolic volume; MI = myocardial infarction; T1 = myocardial longitudinal relaxation time.

LV ejection fraction and mass in a subgroup of 10 randomly chosen patients were independently measured by 2 observers. The intraclass correlation coefficient for reliability of LV ejection fraction was 0.997 (95% CI: 0.963 to 0.999; p < 0.001). The mean absolute difference between measures was 0.99 ml/m^2^, and the root mean square error was 0.93. The intraclass correlation coefficient for reliability of LV mass was 0.997 (95% CI: 0.982 to 0.999; p < 0.001). The mean absolute difference between measures was 2.83 g/m^2^, and the root mean square error was 3.51. Bland-Altman plots showed no evidence of bias.

Baseline infarct size, MVO, and LV function were similar between groups, whereas LV mass was greater in male smokers than male nonsmokers, consistent with the age difference between the groups (Table 1). T2* MRI for myocardial hemorrhage was performed in 286 patients (88%), and 245 (85%) had evaluable T2* maps. The percentage of patients with myocardial hemorrhage was higher in smokers (46%) than in nonsmokers (34%), although this difference was not significant (Table 2).

### Multivariate associations for current smoking with microvascular infarct pathology revealed by MRI

#### Microvascular obstruction

In a binary logistic regression model with baseline characteristics, smoking was independently associated with MVO (OR: 1.72; 95% CI: 1.02 to 2.90; p = 0.041) (Supplemental Appendix), but this association became nonsignificant when infarct size (reflected by peak troponin I concentration) was included in the model (p = 0.11).

#### Myocardial hemorrhage

Smoking was an independent associate of myocardial hemorrhage (OR: 2.55; 95% CI: 1.39 to 4.70; p = 0.003), along with male sex, TIMI coronary flow grade at the end of PCI, and ST-segment resolution (Table 3). Unlike MVO, this association was independent of infarct size, as reflected by peak troponin I (OR: 2.76; 95% CI: 1.42 to 5.37; p = 0.003).

**Table 3:** Multivariate Associations Between Clinical Characteristics, Including Smoking, in 244 Patients With Acute ST-Segment Elevation Myocardial Infarction and Evaluable T2* Mapping for Myocardial Hemorrhage

Binary Logistic Regression	Odds Ratio (95% CI)	p Value
Univariate models		
TIMI coronary flow grade 2/3 pre-PCI	0.26 (0.13–0.51)	<0.001
Peak troponin I, ng/l	1.00 (1.00–1.00)	<0.001
ST-segment elevation resolution post-PCI		
None, ≤30%	3.37 (1.56–7.26)	0.002
Incomplete, 30% to <70%	2.31 (1.30–4.10)	0.004
Killip class II	1.59 (0.87–2.93)	0.14
Killip class III/IV	30.37 (3.93–234.69)	0.001
Male	1.97 (1.04–3.71)	0.037
Current cigarette smoker	1.66 (0.97–2.84)	0.064
History of hypertension	1.50 (0.87–2.59)	0.143
Multivariate model		
TIMI coronary flow grade 2/3 pre-PCI	0.25 (0.12–0.51)	<0.001
Male	2.67 (1.33–5.38)	0.006
Current cigarette smoker	2.55 (1.39–4.70)	0.003
ST-segment elevation resolution post-PCI		
Incomplete, 30% to <70%	2.44 (1.31–4.53)	0.005
None, ≤30%	3.90 (1.69–9.02)	0.001
History of hypertension	1.81 (0.98–3.34)	0.059
Harrel’s C statistic	0.746	

The univariate associations for the patient characteristics at initial presentation and myocardial hemorrhage include those listed in the table and age (1 year), p = 0.74; body mass index (1 kg/m^2^), p = 0.85; history of hypertension, p = 0.14; hypercholesterolemia, p = 0.39; prior angina, p = 0.39; previous MI, p = 0.61; diabetes, p = 0.16; heart rate (1 beat/min), p = 0.20; and sustained ventricular arrhythmia, p = 0.66. All of the univariate characteristics were used to determine the multivariate model. A manual backward selection approach was used to establish the final model using a p value threshold for exclusion of 0.10. When smoking is removed from the model, the C statistic drops from 0.746 to 0.712.

CI = confidence interval; other abbreviations as in Table 1.

### Smoking and LV outcomes at 6 months

#### LV end-diastolic volume and function

A history of smoking was a multivariate associate of LV end-diastolic volume at 6 months (β = 4.85; 95% CI: −0.59 to 10.28; p = 0.08), but the association was not statistically significant. Smoking was not associated with either adverse LV remodeling or LV ejection fraction at 6 months (p = 0.26).

### Microvascular dysfunction and longer term health outcomes

All (n = 324) patients had long-term follow-up data completed. The median duration of follow-up was 4 years (post-discharge censor duration range 3.9 to 4.9 years). Forty-seven patients (15%) died or experienced a first heart failure event during the index hospitalization or post-discharge. These events included 4 cardiovascular deaths, 11 noncardiovascular deaths, 2 deaths of undetermined cause, and 30 episodes of heart failure (Killip class ≥III [n = 28] or defibrillator implantation [n = 2]). Twenty-three patients (7%) died or experienced a first heart failure hospitalization post-discharge. Smoking was a multivariate associate of all-cause death or heart failure events (n = 32 in smokers, n = 15 in nonsmokers; OR: 2.20; 95% CI: 1.07 to 4.54; p = 0.032) (Table 4).

**Table 4:** Relationships for Smoking Status in 324 Subjects and All-Cause Death or First Hospitalization for Heart Failure or Major Adverse Cardiovascular Events During or After the Index Hospitalization Obtained Using Logistic Regression

Associations	Odds Ratio (95% CI)	p Value
All-cause death or first hospitalization for heart failure		
History of myocardial infarction	6.19 (2.40–15.95)	<0.001
History of hypertension	2.53 (1.28–4.98)	0.007
ST-segment elevation resolution post-PCI		
Incomplete, 30% to <70%	3.41 (1.54–7.56)	0.003
None, ≤30%	4.13 (1.55–11.06)	0.005
Current cigarette smoker	2.20 (1.07–4.54)	0.032
MACE		
History of myocardial infarction	5.16 (1.87–14.25)	0.002
Ventricular arrhythmia	5.11 (1.68–15.50)	0.004
TIMI coronary flow grade 2/3 pre-PCI	0.20 (0.07–0.60)	0.004
Current cigarette smoker	2.79 (2.30–5.99)	0.008
ST-segment elevation resolution post-PCI		
Incomplete, 30% to <70%	2.86 (1.27–6.46)	0.011
None, ≤30%	7.28 (2.78–19.02)	<0.001

The median duration of follow-up was 4 years (post-discharge censor duration range: 1,236 to 1,801 days). Forty-seven patients (15%) died or experienced a first heart failure event during the index hospitalization or post-discharge. Forty-nine patients (15%) experienced MACE.

MACE = major adverse cardiovascular event(s); other abbreviations as in Table 1.

Forty-nine patients (15%) experienced MACE during the index hospitalization or post-discharge. These events included 3 cardiovascular deaths, 4 episodes of STEMI, 13 episodes of non-STEMI, and 29 episodes of heart failure (Killip class ≥III [n = 27] or defibrillator implantation [n = 2]). One patient experienced a STEMI and was censored before experiencing an episode of heart failure, hence there were 30 heart failure events for all-cause death or heart failure and 29 for MACE during a median of 4 years of follow-up (n = 35 in smokers, n = 14 in nonsmokers; OR: 2.79; 95% CI: 2.30 to 5.99; p = 0.008) (Table 4).

## Discussion

We have undertaken a large prospective study of smoking status, infarct pathophysiology, and long-term prognosis in patients with acute STEMI. We found that current smoking is associated with a more favorable cardiovascular risk profile at initial presentation (e.g. younger age, fewer patients with hypertension, and higher coronary flow grades at the end of primary PCI), reflecting better procedural outcomes. Contrary to our hypothesis, acute reperfusion injury (revealed invasively by IMR and noninvasively by ST-segment resolution on electrocardiography) was less pronounced in smokers, suggesting initial favorable findings associated with smoking. Subsequent assessments revealed a less favorable risk profile in smokers in the days following the acute event, including more pronounced systemic inflammation on day 1. In multivariate analyses, smoking was independently associated with a 3-fold increased likelihood of myocardial hemorrhage on day 2, independent of infarct size. Finally, current smoking was independently associated with a 2-fold increased risk for all-cause death or heart failure during a median of 4 years of follow-up (similar associations were observed for MACE).

Despite their younger age, the longer-term prognosis of smokers is worse than that of nonsmokers, including for cardiac events. Our findings should dispel the false notion of any favorable associations between smoking and prognosis after acute STEMI.

### New pathophysiological insights into the apparent smoker’s paradox in acute STEMI

Consistent with prior studies 4, 7, 8, 9, 10, 11, 12, 16, we found that smoking was crudely associated with more favorable presenting characteristics (e.g., younger age) and procedure outcomes. In contrast to prior reports 7, 8, 9, 10, 11, 12, smoking was independently associated with increased risk for adverse longer term health outcomes.

**Figure 3:**
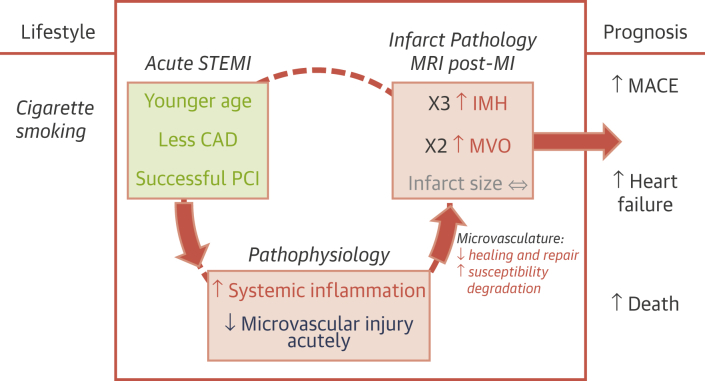
Smoking and Microvascular Pathology After ST-Segment Elevation Myocardial Infarction Schematic of the pathophysiology of cigarette smoking, infarct pathology, and prognosis post–acute ST-segment elevation myocardial infarction (STEMI). The results indicate that smoking is associated with accelerated vascular risk. Despite a typically successful outcome after primary percutaneous coronary intervention (PCI), microvascular pathology within the infarct zone (MVO [microvascular obstruction] and myocardial hemorrhage) is more likely, which increases the long-term risk. CAD = coronary artery disease; IMH = intramyocardial hemorrhage; MACE = major adverse cardiac events.

Our results indicate distinct phases in the early course of MI in smokers (Figure 3). We observed a reverse paradox in that smoking was associated with less reperfusion injury acutely, as revealed by angiography, electrocardiography, and invasive microcirculatory measurements using IMR, but 2 days later, myocardial hemorrhage was more pronounced (Figure 1), even after adjustment for confounding covariates.

We postulate the following explanations for these findings. First, smokers were younger, they had fewer risk factors for microvascular dysfunction 4, 7, 8, 9, 10, 11, 12, and they presented with anterior MI less often. These factors most likely explain why smokers had less reperfusion injury acutely. Given the harmful effects of smoking on vascular health (4), the reperfused microvessels in smokers may have reduced repair potential and thus greater susceptibility to progressive degradation within the infarct core in the days after reperfusion. Second, in a serial imaging study, we found that myocardial hemorrhage was preceded by MVO (18), as MVO is an upstream event that may resolve. Our results indicate that the progression to infarct zone hemorrhage, rather than recovery without hemorrhage, was more likely in smokers than nonsmokers. Even after accounting for infarct size, the association between smoking and infarct zone hemorrhage persisted, unlike for MVO. This observation is prognostically relevant because myocardial hemorrhage reflects irreversible tissue damage 17, 18. A recent study that described the independent prognostic importance of MVO post-MI did not include information on myocardial hemorrhage (42). Using contemporary, multiparametric cardiac magnetic resonance using T2* mapping, we have found that myocardial hemorrhage is a much stronger determinant of adverse prognosis than MVO (18). The lack of an association between smoking and myocardial hemorrhage in prior studies (12) may have been related to the use of dark-blood T2-weighted MRI, which has limited diagnostic accuracy (38) when considered against the more sensitive and specific T2* mapping 18, 35. Third, current smoking was associated with systemic inflammation (Table 1), which is also independently associated with microvascular pathology (18) and prognosis (43). Inflammation may serve as a mechanistic link mediating progressive vascular injury and reduced repair potential within the infarct zone, leading in turn to myocardial hemorrhage. Some confounding observations included lower circulating NT-proBNP concentrations in smokers than in nonsmokers. This may be explained by the fact that compared with nonsmokers, current smokers were younger and had better renal function, and anterior MI occurred less often. Circulating concentrations of troponin are paradoxically lower in current smokers from the general population. The mechanisms of this observation remain to be elucidated (44).

### Relevance to public health

Our study has important public health implications. We observed that during longer term follow-up, current smoking prior to acute STEMI is a multivariate associate of all-cause death or heart failure, providing new insights into the smoker’s paradox. The association between microvascular pathology and smoking provides a mechanistic explanation for the adverse risk. Because previous MI is a strong predictor of recurrent MI, efforts from health care professionals to help patients achieve smoking cessation are all the more relevant.

### Study limitations

We performed a single-center natural history study and enrolled a high proportion (∼90%) of screened patients. The reasons in the majority of those patients not enrolled (Figure 2) related to contraindications to MRI (e.g., claustrophobia) and logistics, rather than the severity of MI, implying that the cohort is representative of an all-comers STEMI population. Smoking status was self-reported. We did not gather information on the duration, frequency, or type of cigarette smoking, nor did we gather information on smoking status during follow-up. All smokers were referred for smoking cessation therapy as part of cardiac rehabilitation. The mean LV ejection fraction (55%) probably reflects the comparatively short ischemic time overall (the median door-to-balloon time in our hospital is 21 min). The study population included 21 patients initially treated with thrombolysis, and 14 of these patients underwent rescue PCI. The main results of our study were unchanged when these patients were removed. Cigarette smoking post-MI may have influenced the prognostic associations between a history of smoking before the initial MI and adverse cardiovascular events in the longer term. Smoking status was not reevaluated at 6 months, which represents a limitation of the study design. The multivariate model (Table 3) could be considered as overfitted by traditional standards. No adjustment was made for skewed variables in the multivariate analyses. Our analysis does not permit inference on causality, and further studies are warranted.

## Conclusions

Current smokers presenting with acute STEMI are nearly 10 years younger than nonsmokers, consistent with an accelerated vascular risk. Current smoking is independently associated with irreversible infarct zone hemorrhage and systemic inflammation following acute STEMI and worse longer term health outcomes.

Perspectives **COMPETENCY IN MEDICAL KNOWLEDGE:** Our study provides new insights into the smoker’s paradox. We have shown that cigarette smoking is independently associated with irreversible infarct zone hemorrhage, systemic inflammation following acute STEMI, and worse longer term health outcomes. Our results should dispel the false notion of any favorable associations between smoking and prognosis after acute STEMI. Because previous MI is a strong predictor of recurrent MI, efforts from health care professionals to help patients achieve smoking cessation are all the more relevant.

**TRANSLATIONAL OUTLOOK:** Future studies are needed to confirm the findings using biochemical indicators of smoking status and using information on the duration, frequency, or type of cigarette smoking, as well as smoking status during follow-up.
